# Phylogenetically diverse bacteria isolated from tattoo inks, an azo dye-rich environment, decolorize a wide range of azo dyes

**DOI:** 10.1186/s13213-021-01648-2

**Published:** 2021-09-21

**Authors:** Seong Won Nho, Xuewen Cui, Ohgew Kweon, Jinshan Jin, Huizhong Chen, Mi Sun Moon, Seong-Jae Kim, Carl E. Cerniglia

**Affiliations:** 1 Division of Microbiology, National Center for Toxicological Research, U.S. Food and Drug Administration, Jefferson, AR, USA.; 2 Present address: Sichuan Institute for Food and Drug Control, Chengdu 611731, Sichuan, China.; 3 Office of Cosmetics and Colors, Center for Food Safety and Applied Nutrition, U.S. Food and Drug Administration, College Park, MD, USA.

**Keywords:** Tattoo ink, Microbial contamination, Bacterial azo dye degradation

## Abstract

**Purpose:**

There has been an interest in the microbial azo dye degradation as an optional method for the treatment of azo dye-containing wastes. Tattoo ink is an extremely unique azo dye-rich environment, which have never been explored in terms of microorganisms capable of degrading azo dyes. Previously, we isolated 81 phylogenetically diverse bacteria, belonging to 18 genera and 52 species, contaminated in tattoo inks. In this study, we investigated if these bacteria, which can survive in the azo dye-rich environment, have an ability to degrade azo dyes.

**Methods:**

We conducted a two-step azo dye degradation (or decolorization) assay. In step 1, a high-throughput degradability assay was done for 79 bacterial isolates using Methyl Red and Orange II. In step 2, a further degradation assay was done for 10 selected bacteria with a representative of 11 azo dyes, including 3 commercial tattoo ink azo dyes. Degradation of azo dyes were calculated from measuring optical absorbance of soluble dyes at specific wavelengths.

**Results:**

The initial high-throughput azo dye assay (step 1) showed that 79 isolates had a complete or partial degradation of azo dyes; > 90% of Methyl Red and Orange II were degraded within 24 h, by 74 and 20 isolates, respectively. A further evaluation of azo dye degradability for 10 selected isolates in step 2 showed that the isolates, belonging to *Bacillus*, *Brevibacillus*, *Paenibacillus*, and *Pseudomonas*, exhibited an excellent decolorization ability for a wide range of azo dyes.

**Conclusions:**

This study showed that phylogenetically diverse bacteria, isolated from azo dye-rich tattoo inks, is able to degrade a diverse range of azo dyes, including 3 azo dyes used in commercial tattoo inks. Some of the strains would be good candidates for future studies to provide a systematic understanding of azo dye degradation mechanisms.

## Background

Azo dyes, which are the most common class of dyes containing one or more azo (–N=N–) bonds, are widely used in textile, paper, cosmetic, pharmaceutical, and food industries (Carmen and Daniela 2012). Reports in the literature indicate that azo dyes and their intermediates may be toxic or mutagenic and carcinogenic, posing negative impact on the environmental health (Chung 2016; Feng et al. 2012). Industries release large amount of insufficiently treated azo dye-containing waste in the environment (Rawat et al. 2016).

Physicochemical-based methods, such as absorption, coagulation, precipitation, chemical transformation, and incineration, have often been used for the remediaion of azo dyes-containing waste effluents in the environment (Robinson et al. 2001). However, these methods have limited applicability due to lack of efficiency, high cost, generation of toxic by-products, and high energy requirements. In recent years, biotechnological methods using microorganisms have been suggested as an option to overcome the limitations of the physicochemical-based approaches for the reduction of azo dyes in the environment (Forgacs et al. 2004; Robinson et al. 2001). Diverse categories of microorganisms, including bacteria, fungi, and algae, have been reported to be capable of degrading a wide range of azo dyes (Solís et al. 2012; Stolz 2001).

Previously, we isolated upwards of 80 bacteria, belonging to 18 genera and 52 species, from a survey study of unopened sealed bottles of tattoo inks for microbial composition (Nho et al. 2020; Nho et al. 2018). Although the survey study was originally initiated to determine if microbial contaminants occurred in the tattoo inks, results of the study led us into a different question; that is, whether the presence of such microorgnaisms has something further to do with tattoo inks, an environment known to be highly abundant with azo dyes (Bäumler et al. 2000). We therefore decided to study whether there was a functional connection between the isolates found in the tattoo inks, which can survive in an azo dye-rich environment, and their ability to biodegrade azo dyes. Here, we present the data on the azo dye degradation (or decolorization) assay that we conducted on these phylogenetically diverse bacterial isolates.

Azo dye-degrading microorganisms have often been isolated from various environments, particularly including textile effluents, activated sludge, or azo dye-contaminated soils and ground waters near textile industries (Gomare and Govindwar 2009; Jin et al. 2007; Meehan et al. 2001; Srinivasan et al. 2014; Telke et al. 2010; Xu et al. 2005). However, to our knowledge, tattoo inks, an extremely unique azo dye-rich environment, have previously never been explored in terms of azo dye degradation. In this study, we examined if these bacterial isolates have an ability of azo dye degradation, and if so, how well can they degrade structurally different kinds of azo dyes.

## Materials and methods

### Dyes and chemicals

**Fig. 1:**
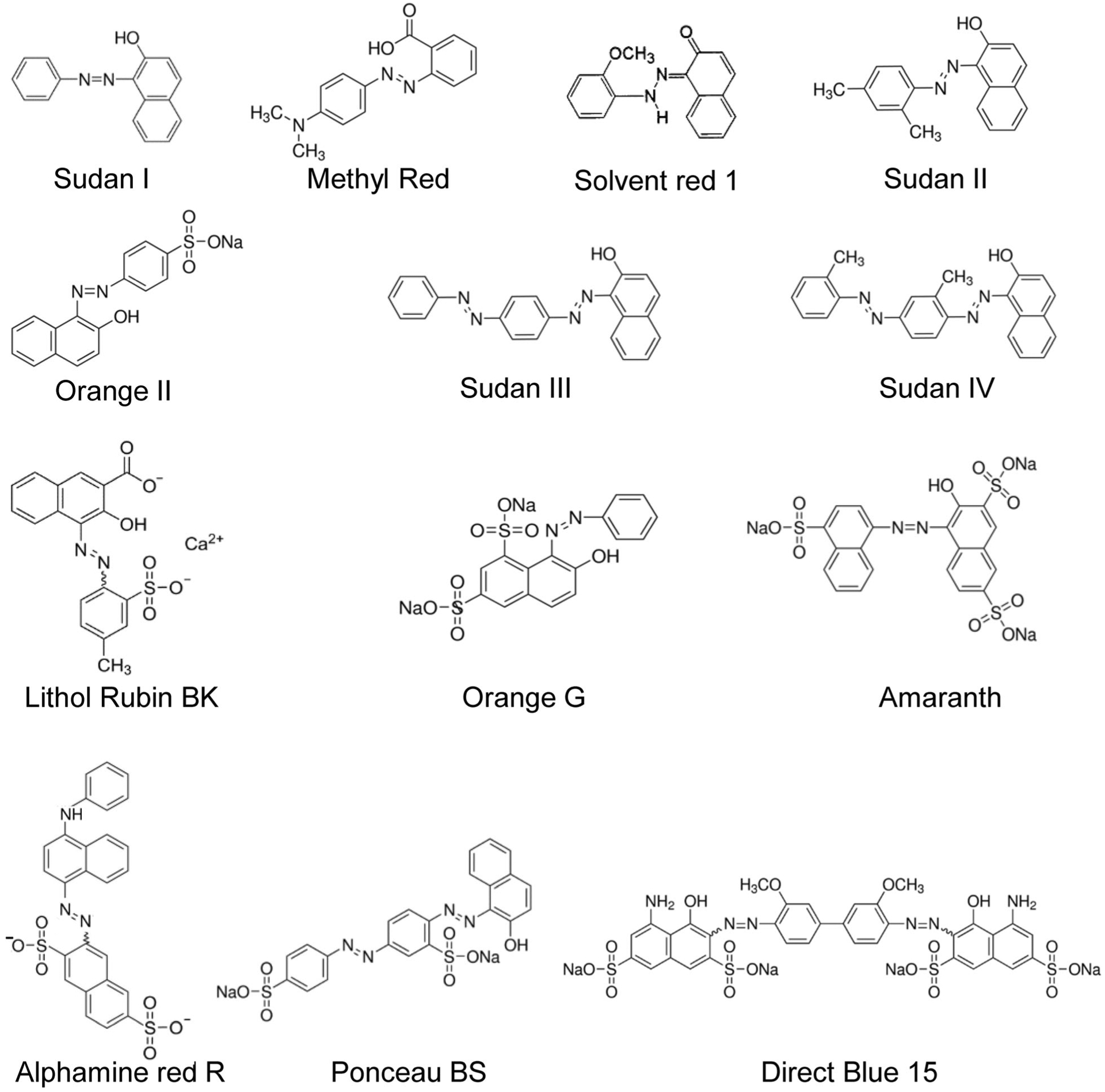
Chemical structures of 13 azo dyes used in this study

Methyl Red, Sudan I, Sudan II, Sudan III, Sudan IV, Toluidine Red, Orange II, Fast Dark Blue R Salt, Lithol Rubin BK, Orange G, Amaranth, Ponceau BS, Direct Blue 15, and dimethyl sulfoxide (DMSO) were purchased from Sigma-Aldrich Inc. (Saint Louis, MO, USA). Solvent Red 1, Hansa Yellow, Hansa Orange, and Alphamine Red R were purchased from Fisher Scientific (Hanover Park, IL, USA). Diarylide Yellow HR (Pigment Yellow 83) was purchased from Santa Cruz Biotechnology Inc. (Dallas, TX, USA). Chemical structures of azo dyes used in this study were shown in Fig. 1. Tryptic Soy Agar (TSA) and Tryptic Soy Broth (TSB) used for bacterial growth were purchased from Fisher Scientific (Hanover Park, IL, USA). Stock solutions of azo dyes (10 mM) were prepared by using 100% DMSO as solvent, then dye solutions were diluted with TSB in degradation assay. Final concentration of DMSO and dye in the assays was 1% and 100 μM except as indicated otherwise.

### Bacterial growth conditions and azo dye decolorization assay using 96-well microphates

All 79 bacterial strains used in this study (Table 1) were isolated from tattoo inks in our previous study (Nho et al. 2018). Bacteria were incubated overnight in TSB at 37 °C with shaking at 250 rpm. The overnight grown bacterial cultures were inoculated into 96-well microplates containing TSB with azo dyes or with 1% DMSO as carrier control. About 5 × 10^7^ – 1 × 10^8^ bacterial cells were inoculated into each well and microplates were incubated at 37 °C without shaking. Optical absorbance was measured for different dyes at specific wavelength (Table 1) at different time points (1 h, 2 h, 4 h, 6 h, 8 h, 18 h, and 24 h) after inoculation using Spectra ax M2 plate reader (Molecular Devices, USA). Calibration curve was determined by measuring optical absorbance of TSB with known concentrations of azo dyes in 96-well microplates. The amount of azo dyes remained in culture media was determined by measuring optical absorbance and calculation with calibration curve. The degradation (%) of azo dyes = 100 − (amount of remain azo dyes/start amount of azo dye) × 100. This experiment was triplicated and repeated twice. The final decolorization (%) is the mean of data from repeated triplicated assay.

### Azo dye decolorization assay using 15-mL conical centrifuge tubes

Selected 10 bacterial species (Table 2) were grown overnight in TSB at 37 °C with shaking at 250 rpm. About 1 × 10^8^ bacterial cells from overnight culture were inoculated into 15-mL conical centrifuge tubes containing 2-mL TSB with azo dye or with 1% DMSO as carrier control, then they were incubated at 37 °C for 48 h without shaking. Bacterial cultures were collected at 48 h by centrifugation at 10,000 rpm for 30 min with Dorval RC6+ Centrifuge (Thermo Scientific). Centrifugation was repeated one more time for the supernatant. Then the supernatants were subjected for measuring optical absorbance of soluble azo dyes at specific wave length (Table 1). To extract insoluble azo dyes, centrifugation pellets were re-suspended with 100% DMSO, and then centrifuged at 10,000 rpm for 10 min. The supernatants of DMSO resuspension were subjected for measuring optical absorbance of insoluble azo dyes. Calibration curve was determined by measuring optical absorbance of known concentrations azo dyes in presence of TSB or 100% DMSO. The amount of soluble azo dyes was calculated with calibration curve determined in the presence of TSB. The amount of insoluble azo dyes was calculated with calibration curve determined in the presence of 100% DMSO. The degradation (%) of azo dyes = 100 − [(amount of soluble azo dye + amount of insoluble azo dye)/initial amount of azo dye] × 100. This experiment was triplicated and repeated twice. The final degradation (%) is the mean of data from repeated triplicated assay.

### Multiple sequence alignment, construction of a phylogenetic tree, and tree annotation

**Fig. 2:**
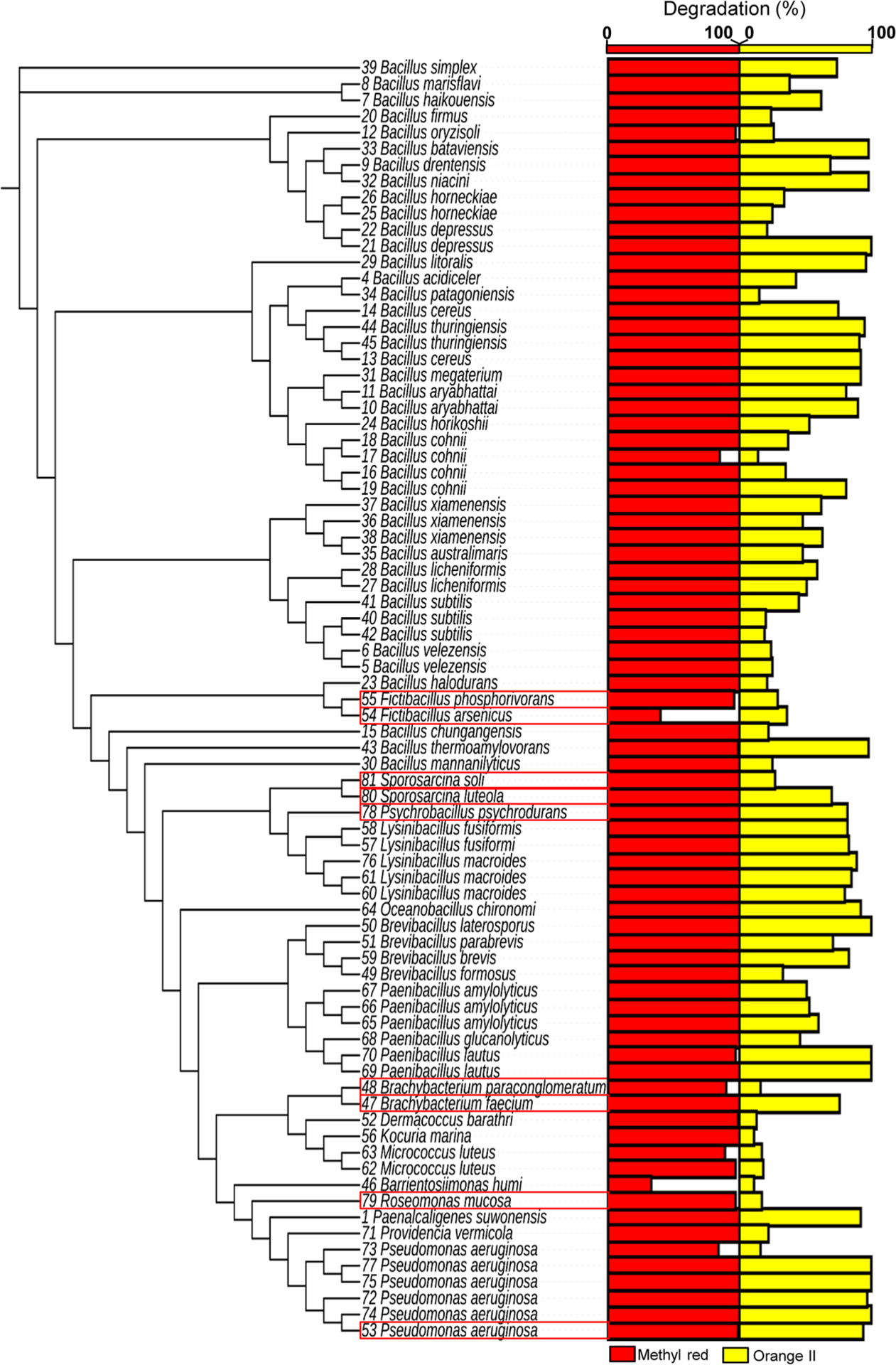
A phylogeny and degradation of Methyl Red and Orange II of 79 diverse bacteria isolated from tattoo inks. Bacterial isolates with a red color rectangle indicate bacterial species with azo dye decolorization ability, which has not been recognized before, and bars (red and yellow) indicate % azo dye degradation of Methyl red and Orange II, respectively

The multiple sequence alignment and construction of a neighbor-joining tree of 16S rDNA sequences from 79 bacterial isolates were performed using Clustal Omega (https://www.ebi.ac.uk/Tools/msa/clustalo/), a new multiple sequence alignment program, that uses seeded guide trees and HMM profile-profile techniques to generate alignments between sequences, with the default settings. To annotate the neighbor-joining tree with degradation rate (%) of Methyl Red and Orange II by 79 bacterial isolates (i.e., Fig. 2, a tree with multi-value bar chart), iTOL (https://itol.embl.de/) was used: input datasets of a neighbor-joining tree in Newick format and a dataset_multibar_template.txt updated with the degradation data of Methyl Red and Orange II by 79 bacterial isolates (Table 1). A template txt file (dataset_multibar_template.txt) can be downloaded from the iTOL website (https://itol.embl.de/help.cgi#datasets).

## Results and discussion

A two-step strategy was used in this study; initially degradation of azo dye was tested for all tattoo ink-derived bacterial isolates using Methyl Red and Orange II (step 1), followed by an evaluation of selected bacteria with respect to the degradation of 11 representative azo dyes, including 3 commercial tattoo ink azo dyes (step 2) (Fig. 1).

### Step 1: Screen 79 bacterial isolates for azo dye degradability using Methyl Red and Orange II

A total of 79 bacterial isolates were tested for the decolorization of azo dyes (Fig. 2 and Table 1). Methyl Red and Orange II were used as model azo dye compounds based on their molecular weight. Overall, results of the screening assay showed that most of these tattoo and PMU ink-derived bacteria well degrade (or decolorize) the low molecular weight azo dye, Methyl Red. Sixty two (78%) and 74 (93%) out of 79 isolates degraded more than 90% of Methyl Red at 8 and 24 h after incubation, respectively (Fig. 2 and Table 1). The remaining five isolates showed degradation of Methyl Red, ranged from 33 to 89% after 24 h incubation. Although there were no previous high-throughput screening data to compare, the results showed that all of the tested bacterial isolates have an ability of azo dye degradation. It indicates that there might be a functional correlation of the capability of azo dye degradation of these bacteria with tattoo inks, an azo dye-rich environment. In case of the high molecular weight azo dye, Orange II, 20 isolates (25%) degraded the dye with > 90% after 24 h incubation while 4 isolates achieved 90% of degradation at 4 h post-incubation (Table 1). The results showed that degradation of Methyl Red was much faster than Orange II, which indicates that the low molecular weight azo dye may be degraded by microorganisms quickly compared with the high molecular weight azo dye (Table 1).

Taxonomically, a wide range of bacterial species were previously reported for their ability to degrade azo dyes, which included bacterial genera *Micrococcus*, *Geobacillus*, *Pseudomonas*, *Kocuria*, *Sphingomonas*, *Bacillus*, *Shewanella*, *Acinetobacter*, *Staphylococcus*, *Aeromonas*, *Corynebacterium*, *Dermacoccus*, and *Kocuria*, to name a few (Saratale et al. 2011; Solís et al. 2012; Stingley et al. 2010). However, in this study, we additionally identified the ability of azo dye degradation in the bacterial genera, *Brachybacterium*, *Desemzia*, *Psychrobacillus*, *Fictibacillus*, *Roseomonas*, and *Sporosarcina*, which have not been previously reported to degrade azo dyes. Generally, isolates which belonged to the same taxonomy in most cases had abilities of azo dye degradation at a comparable level. However, we observed that 4 strains of *B. cohnii* degraded Orange II at different levels, ranged from 14 to 81%. Strains of *B. depressus* and *P. aeruginosa* also showed a significant difference in the level of Orange II degradation (Table 1). It indicated that degradation capability of certain azo dyes depends on the characteristics of each individual strains regardless of its taxonomy.

### Step 2: Evaluation of the selected bacterial isolates for their degradation capability for 11 representative azo dyes, including 3 commercial tattoo ink azo dyes

In step 2, based on the activity of Methyl Red and Orange II degradation in step 1, we selected 10 bacterial isolates. We then further evaluated their ability of azo dye degradation in detail using a broad range of azo dyes (Fig. 1 and Tables 2 and 3). A representative of 11 structurally diverse azo dyes, including 3 commercial tattoo ink azo dyes, was selected for this purpose (Fig. 1). It included, in the order of increasing molecular weight, Sudan I, Solvent Red 1, Sudan II, Sudan III, Sudan IV, Lithol Rubin BK, Orange G, Amaranth, Alphamine Red R, Ponceau BS, and Direct Blue 15. They were selected based on molecular weight (MW 248.3 to 992.8), number of azo bond (1–2 azo bonds), number of benzene ring (2–6 benzene rings), presence of substituted (sulfonated) benzene ring, and the usage as tattoo ink pigments.

The 10 tested bacterial strains belonged to *Bacillus* species (× 4), *Brevi. laterosporus* (× 1), *Paeni. lautus* (× 2), and *P. aeruginosa* (× 3). Overall, all 10 isolates had a varying ability to degrade the 8 structurally diverse representative azo dyes (Fig. 1 and Table 2). The 10 isolates showed a relatively high degradation rate of Sudan I, Amaranth, and Ponceau BS (> 59%) but not for Sudan II and Sudan IV (< 40%). On the other hand, Orange G, Direct Blue 15, and Sudan III were well degraded by some isolates but not well degraded by other isolates. *Paeni. lautus*, *Brevi*. *laterosporus*, and *B. thermoamylovorans* were able to degrade more than 86% of Orange G and Direct Blue 15. On the other hand, only isolates, belonging to *Bacillus* and *Bacillus*-related genera, showed a relatively high degradation (51–85%) toward Sudan III.

Different bacteria have been reported to decolorize azo dyes tested in this study. Skin bacteria belonging to the genera *Staphylococcus*, *Corynebacterium*, *Micrococcus*, *Dermacoccus*, and *Kocuria*, were able to decolorize Methyl Red in nutrient broth within 24 h with 74–100% reduction under static condition (Stingley et al. 2010). In our study, we had strains belonging to *Dermacoccus* and *Kocuria* genera, which had achieved over 99% reduction of Methyl Red in TSB by 24 h (Table 1). A strains of *Brevi. laterosporus*, was found to decolorize 93% of Methyl Red within 12 h in nutrient broth (Gomare and Govindwar 2009). Complete decolorization of Mtehyl Red was observed in our study within 24 h in TSB under static condition by a strain of *Brevi. laterosporus* (Table 1). Among those azo dyes tested in step 2, only one case exists in the literature where a *P. aeruginosa* strain was able to decolorize 96% of Amaranth in distilled water within 6 h (Jadhav et al. 2013). Whereas, in our study, 3 different strains of *P. aeruginosa* were capable of decolorizing Amaranth in TSB with the range of reduction rate between 70 and 89% within 48 h (Table 2).

In case of azo dyes widely used as pigments in the commercial tattoo ink products, the 10 isolates degraded more than 74% of Lithol Rubin BK and Alphamine Red R (Fig. 1 and Table 3). On the other hand, only *Brevi. Laterosporus* (#50) showed a high degradation rate (95%) to Solvent Red 1; other isolates showed a degradation rate around or less than 50% (Fig. 1 and Table 3). There are no reports available on the decolorization of azo dyes used in tattoo inks. Thus, the data obtained in this study may contribute to decolorization of tattoo ink azo dyes by pure bacterial cultures.

Overall, the results of this study suggest that there is a general dependency on azo dye structure and bacterial taxonomy and pleiotropic and epistatic functional interactions among diverse azo dye degrading enzymes in the degradation of azo dyes. Out of the tested 10 isolates, *B. thermoamylovorans* (isolate #43), *Brevi. laterosporus* (#50), and 2 strains of *Paeni. lautus* (#69 and #70) were the top 4 bacteria with high efficiencies of azo dye degradation. High degradation ability of azo dyes by members of the genera *Brevibacillus* and *Paenibacillus* have been shown (Alhassani et al. 2007; Gomare and Govindwar 2009; Nawahwi et al. 2013; Ramya et al. 2008; Srinivasan et al. 2014).

## Conclusions

This study was a novel attempt to (i) examine the capability of azo dye degradation (or decolorization) of bacterial strains isolated from tattoo inks, which provide an azo dye-rich environment, and (ii) introduce a high-throughput azo dye degradation assay to identify azo dye-degrading bacteria. The study confirmed the ability of phylogenetically diverse bacteria, isolated from azo dye-rich tattoo inks, to degrade a diverse range of azo dyes, including 3 azo dyes used in commercial tattoo inks. Four bacterial isolates, belonging to B. thermoamylovorans (#43), Brevi. laterosporus (#50), and Paeni. lautus (#69 and #70), exhibited an excellent capability of degradation of a diverse range of azo dyes. Further studies, including genome sequencing and functional genomics with these isolates, should be followed for a systematic understanding of the mechanism of azo dye degradation in these microorganisms, which is essential for their practical use in bioremediation application for removal of azo dyes via comparative insights on the differences in properties from the azo pigment-degrading bacteria that have been reported so far.

## Figures and Tables

**Table 1:** Degradation (%)of Methyl Red and Orange II by 79 bacterial isolates^a^

Isolate #	Identity based on 16S rRNA	BLAST on NCBI 16S RefSeq database		Methyl Red^b^			Orange II^b^		
		Type strain (Sequence ID)	% Identity	1 h	8 h	24 h	1 h	8 h	24 h
1	*Paenalcaligenes suwonensis*	*P. suwonensis* ABC02-12 (NR_133804.1)	99.23	20 ± 3	96 ± 4	100 ± 0	5 ± 3	18 ± 5	92 ± 8
4	*Bacillus acidiceler*	*B. acidiceler* CBD 119 (NR_043774.1)	99.67	99 ± 1	100 ± 0	100 ± 0	9 ± 1	25 ± 4	43 ± 3
5	*Bacillus velezensis*	*B. velezensis* FZB42 (NR_075005.2)	99.64	74 ± 10	100 ± 0	100 ± 0	8 ± 2	12 ± 1	25 ± 2
6	*Bacillus velezensis*	*B. velezensis* FZB42 (NR_075005.2)	99.74	100 ± 0	100 ± 0	100 ± 0	2 ± 1	8 ± 4	24 ± 2
7	*Bacillus haikouensis*	*B. haikouensis* C-89 (NR_148273.1)	99.39	62 ± 13	100 ± 0	100 ± 0	7 ± 2	13 ± 2	62 ± 5
8	*Bacillus marisflavi*	*B. marisflavi* TF-11 (NR_025240.1)	99.73	100 ± 0	100 ± 0	100 ± 0	5 ± 1	13 ± 1	38 ± 3
9	*Bacillus drentensis*	*B. drentensis* IDA1967 (NR_029002.1)	99.65	30 ± 1	100 ± 0	100 ± 0	7 ± 1	56 ± 10	69 ± 6
10	*Bacillus aryabhattai*	*B. aryabhattai* B8W22 (NR_115953.1)	99.87	89 ± 11	100 ± 0	100 ± 0	4 ± 1	24 ± 6	90 ± 10
11	*Bacillus aryabhattai*	*B. aryabhattai* B8W22 (NR_115953.1)	99.80	24 ± 5	100 ± 0	100 ± 0	10 ± 1	26 ± 4	81 ± 11
12	*Bacillus oryzisoli*	*B. oryzisoli* 1DS3-10 (NR_151979.1)	98.88	89 ± 2	98 ± 2	97 ± 3	7 ± 1	8 ± 2	26 ± 5
13	*Bacillus cereus*	*B. cereus* CCM 2010 (NR_115714.1)	99.74	100 ± 0	100 ± 0	100 ± 0	7 ± 4	42 ± 3	92 ± 8
14	*Bacillus cereus*	*B. cereus* CCM 2010 (NR_115714.1)	99.01	100 ± 0	100 ± 0	100 ± 0	47 ± 5	75 ± 4	75 ± 4
15	*Bacillus chungangensis*	*B. chungangensis* CAU 348 (NR_116709.1)	99.32	15 ± 5	93 ± 8	100 ± 0	9 ± 2	9 ± 2	22 ± 1
16	*Bacillus cohnii*	*B. cohnii* DSM 6307 (NR_026138.1)	99.67	48 ± 5	100 ± 0	100 ± 0	11 ± 1	13 ± 2	35 ± 4
17	*Bacillus cohnii*	*B. cohnii* DSM 6307 (NR_026138.1)	99.40	9 ± 3	41 ± 2	85 ± 4	9 ± 1	10 ± 1	14 ± 1
18	*Bacillus cohnii*	*B. cohnii* DSM 6307 (NR_026138.1)	99.60	21 ± 3	100 ± 0	100 ± 0	8 ± 2	13 ± 2	37 ± 7
19	*Bacillus cohnii*	*B. cohnii* DSM 6307 (NR_026138.1)	99.67	57 ± 12	100 ± 0	100 ± 0	9 ± 3	18 ± 6	81 ± 11
20	*Bacillus firmus*	*B. firmus* IAM 12464 (NR_112635.1)	99.13	96 ± 4	100 ± 0	100 ± 0	8 ± 1	10 ± 2	24 ± 3
21	*Bacillus depressus*	*B. depressus* BZ1 (NR_146034.1)	99.52	50 ± 6	100 ± 0	100 ± 0	9 ± 1	26 ± 1	100 ± 0
22	*Bacillus depressus*	*B. depressus* BZ1 (NR_146034.1)	99.59	18 ± 5	100 ± 0	100 ± 0	7 ± 3	13 ± 1	21 ± 6
23	*Bacillus halodurans*	*B. halodurans* DSM 497 (NR_025446.1)	99.87	18 ± 5	100 ± 0	100 ± 0	7 ± 1	11 ± 2	21 ± 1
24	*Bacillus horikoshii*	*B. horikoshii* DSM 8719 (NR_040852.1)	99.27	25 ± 5	100 ± 0	100 ± 0	8 ± 0	11 ± 1	53 ± 7
25	*Bacillus horneckiae*	*B. horneckiae* 1P01SC (NR_116474.1)	99.34	33 ± 5	100 ± 0	100 ± 0	9 ± 4	11 ± 5	25 ± 11
26	*Bacillus horneckiae*	*B. horneckiae* 1P01SC (NR_116474.1)	99.78	40 ± 5	100 ± 0	100 ± 0	3 ± 2	11 ± 1	34 ± 3
27	*Bacillus licheniformis*	*B. licheniformis* DSM 13 (NR_118996.1)	99.54	41 ± 4	100 ± 0	100 ± 0	8 ± 6	22 ± 4	51 ± 7
28	*Bacillus licheniformis*	*B. licheniformis* DSM 13 (NR_118996.1)	99.54	77 ± 8	99 ± 1	100 ± 0	12 ± 3	20 ± 3	59 ± 4
29	*Bacillus litoralis*	*B. litoralis* SW-211 (NR_043015.1)	98.60	44 ± 12	100 ± 0	100 ± 0	12 ± 4	18 ± 8	96 ± 4
30	*Bacillus mannanilyticus*	*B. mannanilyticus* AM-001 (NR_040851.1)	96.93	30 ± 6	100 ± 0	100 ± 0	10 ± 0	11 ± 1	25 ± 6
31	*Bacillus megaterium*	*B. megaterium* NBRC 15308 (NR_112636.1)	99.86	100 ± 0	100 ± 0	100 ± 0	7 ± 2	22 ± 2	92 ± 8
32	*Bacillus niacini*	*B. niacini* IFO15566 *(NR_024695.1)*	98.94	24 ± 10	100 ± 0	100 ± 0	8 ± 1	50 ± 10	98 ± 1
33	*Bacillus bataviensis*	*B. bataviensis* IDA1115 (NR_036766.1)	97.32	95 ± 5	100 ± 0	100 ± 0	9 ± 3	59 ± 5	98 ± 2
34	*Bacillus acidiceler*	*B. acidiceler* CBD 119 (NR_043774.1)	99.67	42 ± 7	100 ± 0	100 ± 0	7 ± 2	9 ± 2	15 ± 2
35	*Bacillus australimaris*	*B. australimaris* MCCC 1A05787 (NR_148787.1)	99.67	100 ± 0	100 ± 0	100 ± 0	13 ± 3	10 ± 1	48 ± 3
36	*Bacillus xiamenensis*	*B. xiamenensis* MCCC 1A00008 (NR_148244.1)	99.47	100 ± 0	100 ± 0	100 ± 0	7 ± 1	12 ± 2	48 ± 1
37	*Bacillus xiamenensis*	*B. xiamenensis* MCCC 1A00008 (NR_148244.1)	98.81	100 ± 0	100 ± 0	100 ± 0	10 ± 4	12 ± 1	62 ± 1
38	*Bacillus xiamenensis*	*B. xiamenensis* MCCC 1A00008 (NR_148244.1)	99.74	96 ± 4	100 ± 0	100 ± 0	10 ± 1	16 ± 2	63 ± 2
39	*Bacillus simplex*	*B. simplex* NBRC 15720 (NR_042136.1)	99.34	72 ± 8	100 ± 0	100 ± 0	18 ± 2	74 ± 7	74 ± 7
40	*Bacillus subtilis*	*B. subtilis* IAM 12118 (NR_112116.2)	99.60	97 ± 3	100 ± 0	100 ± 0	11 ± 0	11 ± 3	20 ± 3
41	*Bacillus subtilis*	*B. subtilis* IAM 12118 (NR_112116.2)	99.47	89 ± 5	100 ± 0	100 ± 0	8 ± 1	14 ± 1	45 ± 3
42	*Bacillus subtilis*	*B. subtilis* IAM 12118 (NR_112116.2)	99.34	100 ± 0	100 ± 0	100 ± 0	7 ± 2	8 ± 1	19 ± 3
43	*Bacillus thermoamylovorans*	*B. thermoamylovorans* LMG 18084 (NR_117028.1)	99.60	19 ± 5	98 ± 2	99 ± 1	13 ± 3	97 ± 3	98 ± 1
44	*Bacillus thuringiensis*	*B. thuringiensis* IAM 12077 (NR_043403.1)	99.87	100 ± 0	100 ± 0	100 ± 0	12 ± 0	56 ± 3	95 ± 2
45	*Bacillus thuringiensis*	*B. cereus* CCM 2010 (NR_115714.1)	99.80	96 ± 4	100 ± 0	100 ± 0	4 ± 2	44 ± 9	91 ± 5
46	*Barrientosiimonas humi*	*B. humi* 39 (NR_126227.1)	99.80	13 ± 1	18 ± 1	33 ± 2	9 ± 2	9 ± 2	11 ± 2
47	*Brachybacterium faecium*	*B. faecium* DSM 4810 (NR_074655.2)	97.78	14 ± 2	100 ± 0	100 ± 0	11 ± 1	13 ± 3	76 ± 12
48	*Brachybacterium paraconglomeratum*	*B. paraconglomeratum* LMG 19861 (NR_025502.1)	99.12	14 ± 5	44 ± 8	90 ± 7	7 ± 2	10 ± 2	16 ± 5
49	*Brevibacillus formosus*	*B. formosus* DSM 9885 (NR_040979.1)	99.32	36 ± 4	100 ± 0	100 ± 0	10 ± 2	13 ± 1	33 ± 2
50	*Brevibacillus laterosporus*	*B. laterosporus* DSM 25 (NR_112212.1)	99.53	82 ± 8	77 ± 0	100 ± 0	9 ± 1	99 ± 1	100 ± 0
51	*Brevibacillus parabrevis*	*B. parabrevis* IFO 12334 (NR_040981.1)	99.26	28 ± 10	83 ± 6	100 ± 0	9 ± 1	14 ± 2	71 ± 3
52	*Dermacoccus barathri*	*D. barathri* MT2.1 (NR_043261.1)	99.79	14 ± 1	46 ± 4	99 ± 1	9 ± 2	11 ± 1	13 ± 2
53	*Pseudomonas aeruginosa*	*P. aeruginosa* DSM 50071 (NR_117678.1)	99.93	20 ± 5	91 ± 10	99 ± 1	10 ± 5	21 ± 10	94 ± 6
54	*Fictibacillus arsenicus*	*F. phosphorivorans* Ca7 (NR_118455)	99.38	24 ± 5	30 ± 3	40 ± 3	13 ± 3	16 ± 2	36 ± 10
55	*Fictibacillus phosphorivorans*	*F. phosphorivorans* Ca7 (NR_118455)	99.58	31 ± 9	94 ± 6	96 ± 3	9 ± 1	10 ± 3	29 ± 9
56	*Kocuria marina*	*K marina* KMM 3905 (NR_025723.1)	99.86	9 ± 4	54 ± 8	100 ± 0	7 ± 4	9 ± 2	11 ± 2
57	*Lysinibacillus fusiformis*	*L. fusiformis* DSM 2898 (NR_042072.1)	99.93	32 ± 8	100 ± 0	100 ± 0	9 ± 3	11 ± 3	83 ± 4
58	*Lysinibacillus fusiformis*	*L. fusiformis* DSM 2898 (NR_042072.1)	99.80	74 ± 11	100 ± 0	100 ± 0	4 ± 1	10 ± 3	82 ± 8
59	*Lysinibacillus fusiformis*	*L. fusiformis* DSM 2898 (NR_042072.1)	99.32	29 ± 10	100 ± 0	100 ± 0	8 ± 3	12 ± 2	83 ± 4
60	*Lysinibacillus macroides*	*L macroides* LMG 18474 (NR_114920.1)	99.40	44 ± 7	100 ± 0	100 ± 0	8 ± 3	12 ± 3	80 ± 3
61	*Lysinibacillus macroides*	*L macroides* LMG 18474 (NR_114920.1)	99.33	38 ± 8	100 ± 0	100 ± 0	5 ± 2	8 ± 3	85 ± 2
62	*Micrococcus luteus*	*M. luteus* NCTC 2665 (NR_075062.2)	99.53	8 ± 2	42 ± 7	97 ± 3	6 ± 1	11 ± 3	18 ± 3
63	*Micrococcus luteus*	*M. luteus* NCTC 2665 (NR_075062.2)	98.05	10 ± 2	34 ± 5	89 ± 5	7 ± 1	9 ± 2	17 ± 2
64	*Oceanobacillus chironomi*	*O. chironomi* T3944D (NR_043700.1)	97.14	24 ± 6	100 ± 0	100 ± 0	13 ± 4	87 ± 9	92 ± 8
65	*Paenibacillus amylolyticus*	*P. amylolyticus* NRRL NRS-290 (NR_025882.1)	99.27	94 ± 6	100 ± 0	100 ± 0	1 ± 0	17 ± 6	60 ± 2
66	*Paenibacillus tundrae*	*P. tundrae* A10b (NR_044525.1)	99.27	100 ± 0	100 ± 0	100 ± 0	4 ± 1	15 ± 4	53 ± 1
67	*Paenibacillus tundrae*	*P. tundrae* A10b (NR_044525.1)	99.67	89 ± 9	100 ± 0	100 ± 0	6 ± 2	12 ± 2	51 ± 9
68	*Paenibacillus glucanolyticus*	*P. glucanolyticus* DSM 5162 (NR_040883.1)	99.47	16 ± 1	100 ± 0	100 ± 0	8 ± 2	10 ± 1	46 ± 3
69	*Paenibacillus lautus*	*P. lautus* JCM 9073 (NR_040882.1)	99.47	96 ± 4	100 ± 0	100 ± 0	6 ± 4	100 ± 0	100 ± 0
70	*Paenibacillus lautus*	*P. lautus* JCM 9073 (NR_040882.1)	99.60	19 ± 3	96 ± 4	97 ± 4	10 ± 1	100 ± 0	100 ± 0
71	*Providencia vermicola*	*P. vermicola* OP1 (NR_042415.1)	99.66	44 ± 10	100 ± 0	100 ± 0	7 ± 4	9 ± 2	22 ± 2
72	*Pseudomonas aeruginosa*	*P. aeruginosa* DSM 50071 (NR_117678.1)	99.73	11 ± 2	29 ± 2	100 ± 0	11 ± 2	12 ± 2	97 ± 2
73	*Pseudomonas aeruginosa*	*P. aeruginosa* DSM 50071 (NR_117678.1)	99.87	10 ± 3	41 ± 3	84 ± 7	7 ± 1	11 ± 0	16 ± 5
74	*Pseudomonas aeruginosa*	*P. aeruginosa* DSM 50071 (NR_117678.1)	99.93	7 ± 2	28 ± 6	100 ± 0	7 ± 1	14 ± 4	100 ± 0
75	*Pseudomonas aeruginosa*	*P. aeruginosa* DSM 50071 (NR_117678.1)	99.87	9 ± 3	31 ± 9	100 ± 0	11 ± 1	18 ± 5	100 ± 0
76	*Lysinibacillus macroides*	*L macroides* LMG 18474 (NR_114920.1)	99.33	51 ± 3	100 ± 0	100 ± 0	7 ± 4	13 ± 1	89 ± 3
77	*Pseudomonas aeruginosa*	*P. aeruginosa* DSM 50071 (NR_117678.1)	99.87	6 ± 1	28 ± 6	100 ± 0	4 ± 1	15 ± 3	100 ± 0
78	*Psychrobacillus lasiicapitis*	*P. lasiicapitis* NEAU-3TGS17 (NR_159144.1)	98.95	16 ± 2	92 ± 8	100 ± 0	5 ± 2	13 ± 2	82 ± 13
79	*Roseomonas mucosa*	*R. mucosa* MDA5527 (NR_028857.1)	99.72	13 ± 3	38 ± 5	97 ± 2	10 ± 2	9 ± 2	17 ± 3
80	*Sporosarcina luteola*	*S. luteola* Y1 (NR_112844.1)	99.67	11 ± 2	71 ± 7	100 ± 0	5 ± 2	11 ± 3	70 ± 14
81	*Sporosarcina soli*	*S. soli* I80 (NR_043527.1)	99.66	9 ± 1	90 ± 7	100 ± 0	12 ± 1	12 ± 2	27 ± 8

a The data show the mean of degradation (%) ± SD from repeated triplicated assay using 96-well microphates

b 100 μM Orange II and 100 μM Methyl Red were used in the degradation assay, except that 50 μM and 25 μM Methyl Red were used for isolates #4 and #16, respectively and 12.5 μM Methyl Red was used for isolates #12 and #20. Absorbance wavelengths for Methyl Red and Orange II were 430 nm and 475 nm, respectively

**Table 2:** Degradation (%) of 8 structurally diverse azo dyes within 48 h by selected bacterial isolates

Azo dyes	MW	μ^a^	nm^b^	Azo dye degradation (%) in bacterial strains (isolate #)^c^									
				*B. depressus* (#21)	*B. niacini* (#32)	*B. bataviensis* (#33)	*B. thermosamylovorans* (#43)	*Brevi. laterosporus* (#50)	*Paeni. lautus* (#69)	*Paeni. lautus* (#70)	*P. aeruginosa* (#74)	*P. aeruginosa* (#75)	*P. aeruginosa* (#77)
Sudan I	248.3	100	475	86 ± 5	88 ± 2	84 ± 3	85 ± 1	87 ± 1	87 ± 5	87 ± 1	88 ± 5	87 ± 7	90 ± 2
Sudan II	276.3	75	493	39 ± 2	38 ± 1	28 ± 2	40 ± 1	39 ± 6	37 ± 1	39 ± 0	42 ± 1	40 ± 3	46 ± 0
Sudan III	352.4	45	510	77 ± 1	67 ± 1	71 ± 2	51 ± 2	55 ± 7	85 ± 7	71 ± 9	34 ± 1	32 ± 8	29 ± 8
Sudan IV	380.5	45	520	25 ± 1	32 ± 3	39 ± 9	32 ± 0	26 ± 4	40 ± 6	34 ± 6	20 ± 7	21 ± 4	32 ± 1
Orange G	452.4	100	475	16 ± 7	6 ± 1	26 ± 6	97 ± 1	94 ± 2	97 ± 3	97 ± 1	36 ± 4	35 ± 5	36 ± 2
Amaranth	452.4	45	520	59 ± 8	72 ± 1	59 ± 9	99 ± 1	99 ± 1	99 ± 2	99 ± 0	70 ± 1	85 ± 1	89 ± 8
Ponceau BS	556.5	100	510	94 ± 1	92 ± 1	90 ± 6	94 ± 4	92 ± 1	96 ± 2	97 ± 1	97 ± 1	96 ± 1	96 ± 1
Direct Blue 15	992.8	45	620	23 ± 2	23 ± 6	21 ± 5	93 ± 0	86 ± 4	96 ± 0	97 ± 1	39 ± 5	37 ± 7	35 ± 3

a Concentration of azo dyes

b Absorbance wavelength measured for respective azo dyes

c The data show the mean of degradation (%) ± SD from repeated triplicated assay using 15-mL concical centrifuge tubes

**Table 3:** Degradation (%) of 3 azo dyes used widely as pigments in the commercial tattoo ink products by selected bacterial isolates

				Azo dye degradation (%) in bacterial strains (isolate #)^c^									
Azo dyes	MW	μM^a^	nm^b^	*B. depressus* (#21)	*B. niacini* (#32)	*B. bataviensis* (#33’)	*B. thermosamylovorans* (#43)	*Brevi. laterosporus* (#50)	*Paeni. lautus* (#69)	*Paeni. lautus* (#70)	*P. aeruginosa* (#74)	*P. aeruginosa* (#75)	*P. aeruginosa* (#77)
Solvent Red 1	278.3	100	500	46 ± 9	58 ± 9	47 ± 13	53 ± 8	95 ± 6	53 ± 15	58 ± 18	47 ± 18	45 ± 15	39 ± 15
Lithol Rubin BK	430.3	100	500	99 ± 1	99 ± 1	97 ± 2	81 ± 19	96 ± 3	98 ± 2	100 ± 0	79 ± 9	76 ± 5	74 ± 9
Alphamine Red R	533.6	100	475	92 ± 9^d^	97 ± 1	98 ± 2	94 ± 0	99 ± 1^d^	100 ± 0^d^	98 ± 4^d^	89 ± 2	88 ± 2	88 ± 6

a Concentration of azo dyes

b Absorbance wavelength measured for respective azo dyes

c The data show the mean of degradation (%) ± SD from repeated triplicated assay using 96-well microphates

d Tested with 50 μM of Alphamine Red R

## Data Availability

Data is available under request.
